# MAK33 antibody light chain amyloid fibrils are similar to oligomeric precursors

**DOI:** 10.1371/journal.pone.0181799

**Published:** 2017-07-26

**Authors:** Manuel Hora, Riddhiman Sarkar, Vanessa Morris, Kai Xue, Elke Prade, Emma Harding, Johannes Buchner, Bernd Reif

**Affiliations:** 1 Munich Center for Integrated Protein Science (CIPS-M) at Department Chemie, Technische Universität München (TUM), Germany; 2 Helmholtz-Zentrum München, Deutsches Forschungszentrum für Gesundheit und Umwelt (HMGU), Neuherberg, Germany; University of Pittsburgh School of Medicine, UNITED STATES

## Abstract

Little structural information is available so far on amyloid fibrils consisting of immunoglobulin light chains. It is not understood which features of the primary sequence of the protein result in fibril formation. We report here MAS solid-state NMR studies to identify the structured core of κ-type variable domain light chain fibrils. The core contains residues of the CDR2 and the β-strands D, E, F and G of the native immunoglobulin fold. The assigned core region of the fibril is distinct in comparison to the core identified in a previous solid-state NMR study on AL-09 by Piehl at. al, suggesting that V_L_ fibrils can adopt different topologies. In addition, we investigated a soluble oligomeric intermediate state, previously termed the alternatively folded state (AFS), using NMR and FTIR spectroscopy. The NMR oligomer spectra display a high degree of similarity when compared to the fibril spectra, indicating a high structural similarity of the two aggregation states. Based on comparison to the native state NMR chemical shifts, we suggest that fibril formation via domain-swapping seems unlikely. Moreover, we used our results to test the quality of different amyloid prediction algorithms.

## Introduction

Antibody light chain amyloidosis (AL amyloidosis) is a rare disease caused by amyloid formation of immunoglobulin light chains (LCs) [1,2]. An underlying B-cell dyscrasia causes overproduction and secretion of LCs. In the case of an aggregation-prone LC sequence, this can result in formation of oligomeric intermediates and further of fibrils, which deposit in the inner organs, causing systemic amyloidosis. Heart failure is the dominant cause of death [3,4]. The 4-year overall-survival rate is on the order of 33% [5].

Non-fibrillar oligomers have aroused interest of the research community due to their cytotoxicity as well as their role as folding intermediates [6]. Oligomers have been reported for several amyloid-forming proteins and peptides [7]. In many cases, these oligomers appear to be the most cytotoxic species [8,9]. The mechanism of cytotoxicity is likely due to membrane pore formation [10,11]. Nonetheless, fibrils also exhibit toxicity [12]. In AL amyloidosis, both oligomers and fibrils have been found to be cytotoxic, affecting the cardiomyocyte metabolism [13,14].

Despite the relevance of LC oligomers and fibrils for disease, little is known about their structures. AL fibrils are composed of proteins containing mostly variable domain (V_L_) residues [1]. However, aggregates can also contain LC protein including constant domain (C_L_) residues [1]. The N-terminal part of the V_L_ domain seems to be structured in the fibrils [15,16]. Cryo-EM has been used to determine the steric zipper structure of an AL-protein derived 12-residue peptide fibril [17]. Recently, magic-angle-spinning solid-state NMR (MAS ssNMR) chemical shift assignments and a secondary structure analysis were reported for the κI sequence AL-09 [18,19].

We report here a MAS ssNMR spectroscopic analysis of amyloid fibrils and oligomers formed by the V_L_ domain of MAK33. The murine κ-type V_L_ domain of MAK33 has been studied extensively regarding its native structure [20], biophysical properties and folding pathways [21,22]. Several point mutations facilitate fibril formation [23–26]. We employed the amyloidogenic mutant MAK33 V_L_ S20N [25] to study amyloid fibrils with MAS ssNMR. We were able to identify the structured hydrophobic core region, which comprises mainly residues 60–87. Oligomeric intermediates tend to be short-lived and thus difficult to investigate. In contrast, the MAK33 V_L_ domain forms stable high-molecular weight oligomers at pH 2, which were previously referred to as alternatively folded state (AFS) [21]. These non-fibrillar oligomers were studied with MAS ssNMR, Fourier-transform infrared spectroscopy (FTIR) and transmission electron microscopy (TEM). MAS ssNMR revealed an astonishing similarity of the oligomer and the fibril chemical shifts, indicating a high degree of structural similarity. In contrast, secondary chemical shifts of the fibrils show no correlation with those of the native state, suggesting that fibril formation does not proceed via a domain swapping mechanism.

## Results

**Fig 1:**
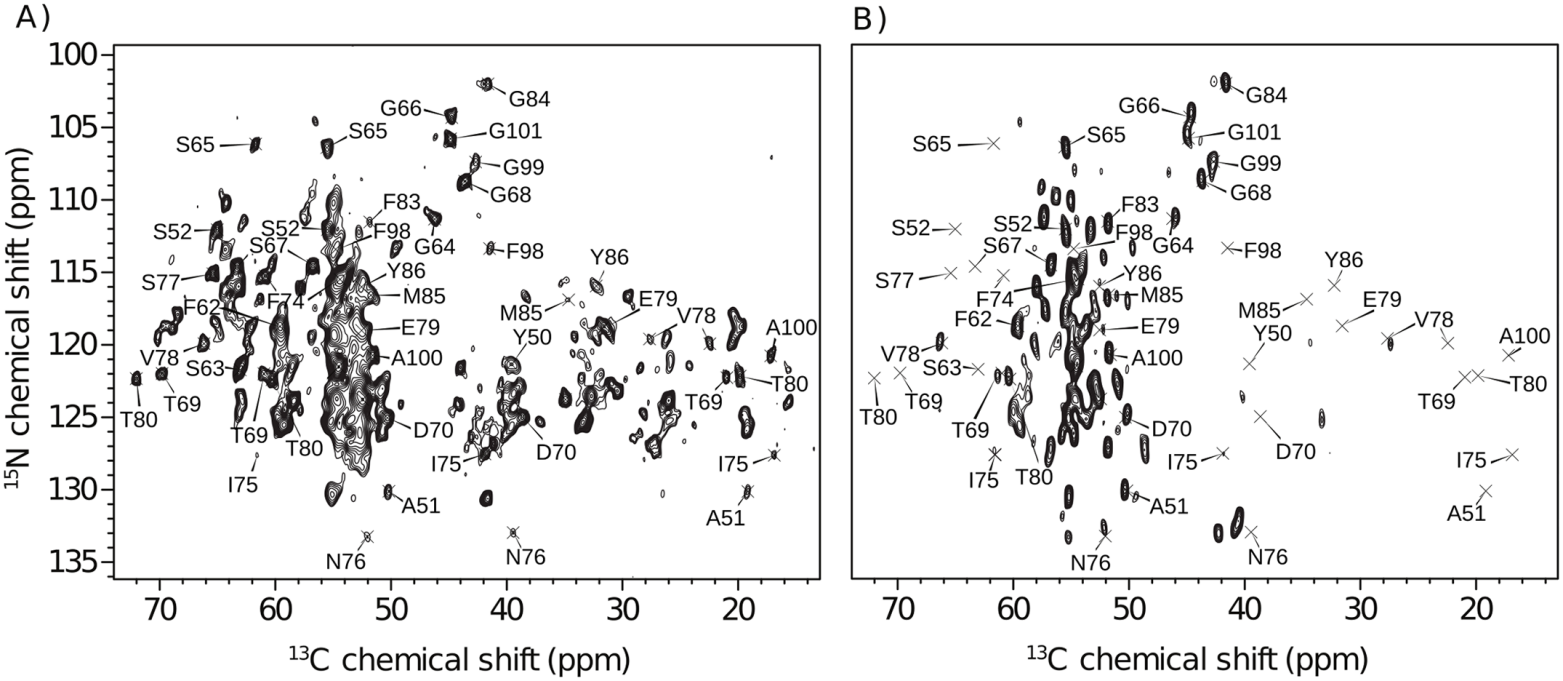
^13^C,^15^N correlations of MAK33 V_L_ S20N fibrils. A) N(CA)CX spectrum of a u-^13^C,^15^N labeled sample. B) NCA spectrum of a 2-^13^C-glycerole isotope labeled sample. Peak positions are identical in A) and B), indicating good reproducibility. The resolution in B) is increased due to sparse isotope labeling.

In order to investigate AL fibril structure, we employed seeded fibrils of the MAK33 V_L_ S20N protein. Two independent fibril preparations yielded highly reproducible MAS ssNMR spectra (Fig 1). Fig 1A shows a 2D-N(CA)CX correlation spectrum obtained from uniformly ^13^C,^15^N labeled protein and Fig 1B a 2D-NCA spectrum for a ^13^C-spin dilute sample. Due to sparse ^13^C labeling of the 2-^13^C-glycerole labeled sample, fewer cross peaks were obtained [27]. Except for this observation, however, the two preparations produced identical NMR spectra. In Fig 1B, assignments were transferred directly from the spectrum of the uniformly ^13^C labeled sample without moving the position of the crosses. Considering the common problem of fibril polymorphism [28], such reproducibility is an important prerequisite for further structural analysis.

The assignment of the structured parts of the MAK33 V_L_ S20N fibrils was accomplished using a variety of MAS ssNMR experiments: PDSD [29], NCACX, NCOCX [30], NCACB, N(CO)CACB, NCOCA and CANCO [31]. A detailed list of all experimental settings is given in the Supporting Information (S1 Table). In total, 36 residues were assigned sequentially, comprising residues 49–52 (located in the complementarity determining region 2 (CDR2) of the native state), residues 60–87 (β-strands D, E and F) and residues 98–101 (β-strand G). For all these residues, only a single set of resonances was observed, indicating that the preparations contained only one fibril polymorph. Chemical shift assignments were deposited in the BioMagResBank (BMRB ID 27065). The ^13^C and ^15^N linewidths were both on the order of 1 ppm at a 750 MHz spectrometer. In particular, the ^15^N transverse relaxation time of 9 ms indicated a high quality of the sample, i.e. a high degree of homogeneity. Representative strip plots of the assignment are shown in S1 Fig.

**Fig 2:**
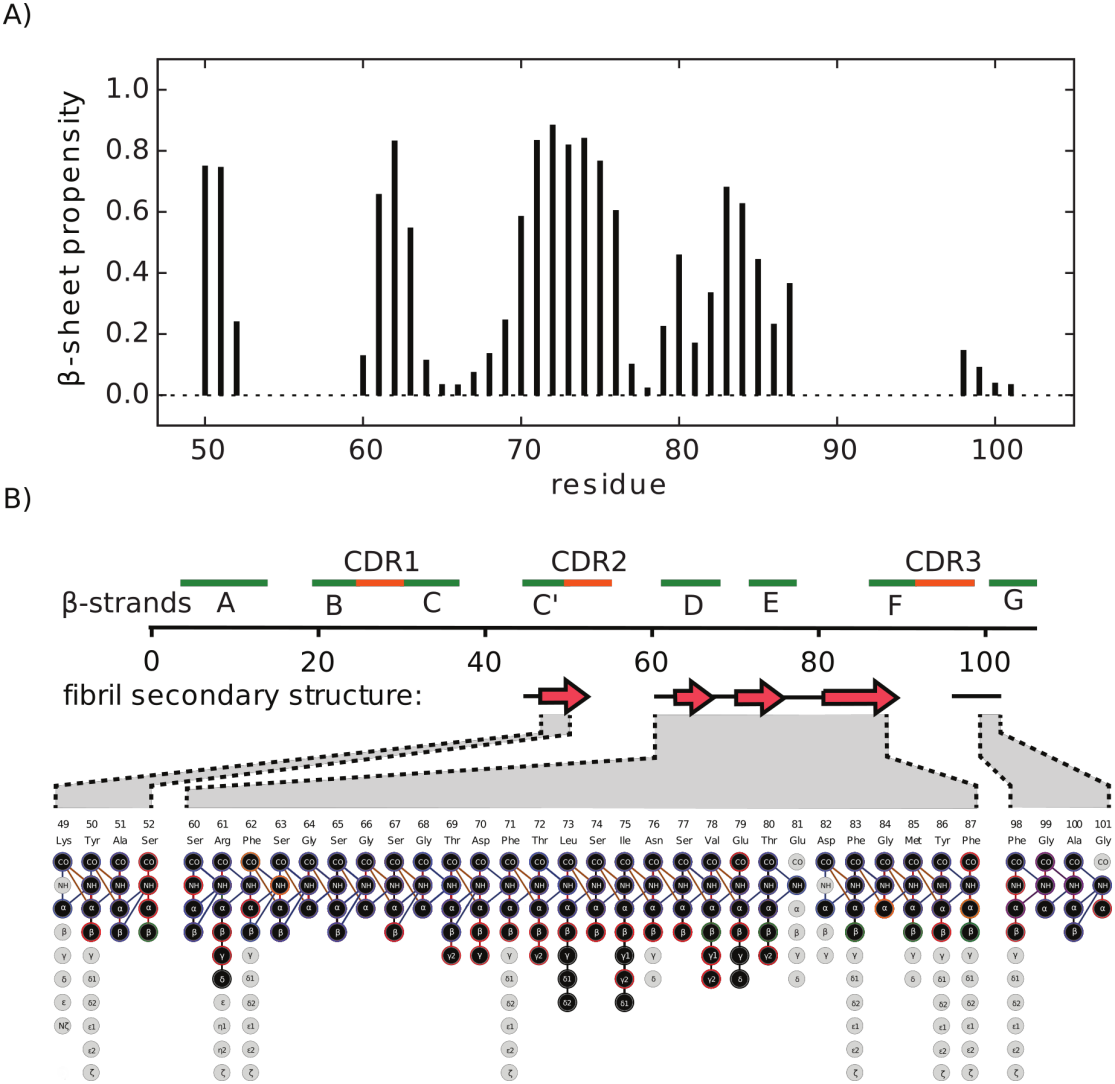
Secondary structure analysis of MAK33 V_L_ in the fibril state. A) β-sheet propensity calculated with TALOS+ [32]. B) Sequence and secondary structure elements of the native V_L_ fold. Green and red bars indicate β-strands and CDRs of the native structure, respectively. Red arrows below the sequence indicate β-strands in the fibril state. The expansion shows the assigned atoms in the aggregated state.

The secondary chemical shift analysis indicates that MAK33 V_L_ S20N populates mostly β-strands in the fibril state, as expected for amyloid fibers. Using TALOS+ [32], we identified four β-strands, with regions of random coil conformation in between them (Fig 2, for Cα, Cβ and CO secondary chemical shifts, see S2 Fig). Twenty additional spin systems could be observed, but not assigned, due to low signal intensities. As signal intensity correlates with rigidity in dipolar-based MAS ssNMR experiments, we assumed that these twenty residues were less structured and located in the flanking region of the core structure.

Dynamic residues cannot be observed with the dipolar-based MAS ssNMR experiments employed here. For complementarity, we conducted an INEPT experiment based on scalar couplings, which is suited to detect highly flexible residues. The resulting spectrum did not yield observable cross peaks (S3 Fig). Therefore, we assume that the remaining residues exhibit dynamics on an intermediate time scale, which can be observed neither with INEPT nor with dipolar-coupling based experiments. Alternatively, the respective residues might be structurally heterogeneous.

**Fig 3:**
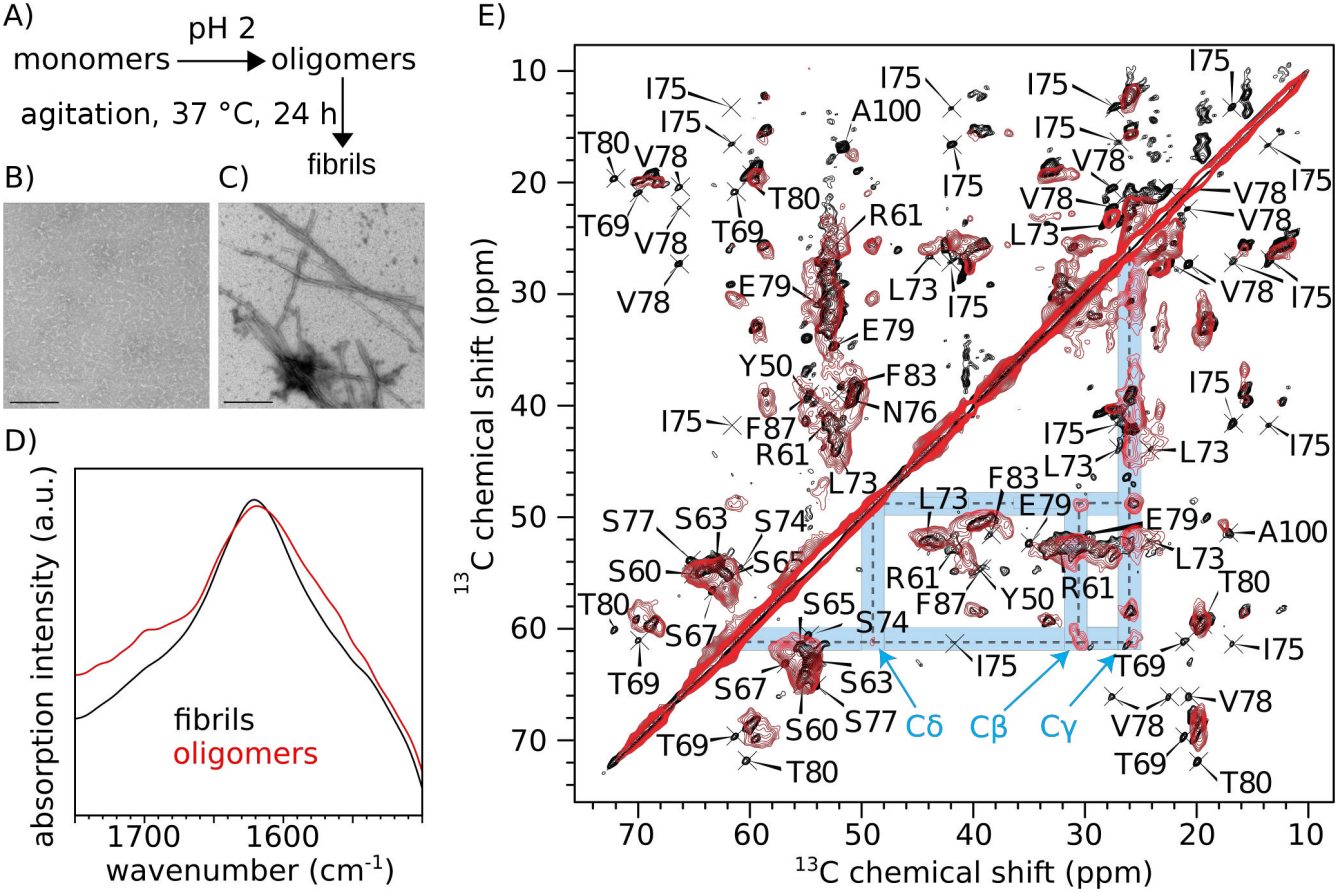
Comparison of MAK33 V_L_ oligomers and fibrils. A) Procedure to form oligomers and fibrils. B), C) Electron micrographs of MAK33 V_L_ S20N oligomers (B) and fibrils (C). The scale bar denotes 200 nm. D) FTIR spectra of MAK33 V_L_ S20N oligomers and fibrils. The peak maxima were 1619 cm^-1^ (oligomers) and 1621 cm^-1^ (fibrils), respectively. The oligomer spectrum displayed an additional peak at 1697 cm^-1^. E) PDSD ^13^C,^13^C-intraresidue correlations of MAK33 V_L_ S20N fibrils and MAK33 V_L_ WT oligomers. The proline spin system, which is more intense in the oligomers, is highlighted in blue.

Having identified the core of the fibrils, we aimed at a comparison with the oligomeric intermediates that are formed during aggregation. While oligomers are typically short-lived and thus difficult to study, MAK33 V_L_ forms stable oligomers at acidic pH (Fig 3A) [21]. An electron microscopic analysis of the oligomers revealed an irregular morphology, but a rather homogeneous size distribution (Fig 3B). The mean diameter of the particles was 9.9 ± 1.5 nm, suggesting in average 30 monomers per oligomer (S4 Fig). This is in excellent agreement with previous studies using analytical ultracentrifugation, which yielded 17 to 42 monomers per oligomer [22]. Upon shaking at 37°C, the oligomers transform to well-structured fibrils within 24 hours (Fig 3C). Using FTIR (Fig 3D), both samples displayed similar peaks, with maxima at 1619 cm^-1^ (oligomers) and 1621 cm^-1^ (fibrils). Amyloid fibrils yield characteristic FTIR maxima between 1611 cm^-1^ and 1630 cm^-1^, whereas peaks of native β-sheet proteins are typically found in the range from 1630 cm^-1^ to 1643 cm^-1^ [33,34]. Hence, the oligomers differ from the native state and resemble the fibrils at the level of secondary structure. The oligomer FTIR spectrum also displayed a less pronounced peak at 1697 cm^-1^. This peak is characteristic for oligomers [35].

We employed MAS solid-state NMR to investigate the V_L_ oligomer structure. Using FROSTY-NMR [36], a PDSD ^13^C,^13^C-correlation of the MAK33 V_L_ WT oligomers was acquired. The similarity between the oligomer and the fibril spectrum is striking (Fig 3E). The chemical shift patterns for many spin systems were very similar. The oligomer resonances were broader in general, which is in good agreement with a less well-defined structure. Some fibril resonances are missing in the oligomer spectrum, which is presumably due to dynamics, resulting in reduced sensitivity. Spectral differences are expected, since oligomers and fibrils show clearly distinct morphologies. The resonances of residues I75, S77, V78, E79 and T80 are missing in the oligomer spectrum and the presence of N76 is unclear due to spectral overlap. In the fibrils, this region corresponds to a loop connecting two β-strands (Fig 2). In the oligomers, this loop might adopt a different conformation or experiences a different chemical environment.

Nevertheless, the similarity of both spectra indicates that the secondary and tertiary structures of the oligomeric and the fibril state are highly similar. We could identify only one spin system, which clearly has a higher signal intensity in the oligomer state than in the fibril state. This spin system corresponded to a proline residue. The proline is presumably in a trans conformation, as the Cβ and Cγ shifts were approximately 30.3 ppm and 25.5 ppm, respectively [37]. MAK33 V_L_ contains five prolines, at positions 8, 15, 44, 59 and 95. However, due to lack of sequential connectivities, we could not assign this proline spin system to one of the five prolines in the primary structure of MAK33 V_L_. Considering which regions of the sequence were assigned, it is likely to be either P44, P59 or P95. The change in NMR signal intensity of this residue suggests that the dynamics of this proline residue increase when the oligomer structure is converted into the fibril state. Proline isomerisation is critical during the initial folding of MAK33 V_L_ [38,39]. Prolines have been reported previously as important switches for amyloid formation in the case of β2-microglobulin [40]. In the case of the amyloidogenic V_L_ domain AL-103, the presence of two consecutive prolines at positions 95 and 95a affects both the kinetic stability and fibril formation [41]. Further analysis of the proline isomerisation states are needed to yield a better understanding of their role in AL amyloid formation.

## Discussion

Here we reported on the structured core residues of an immunoglobulin V_L_-κ sequence in the amyloid fibril and oligomeric state. Considering the tremendous variability of antibody sequences, it is interesting whether these results are transferable to other light chain sequences.

**Fig 4:**
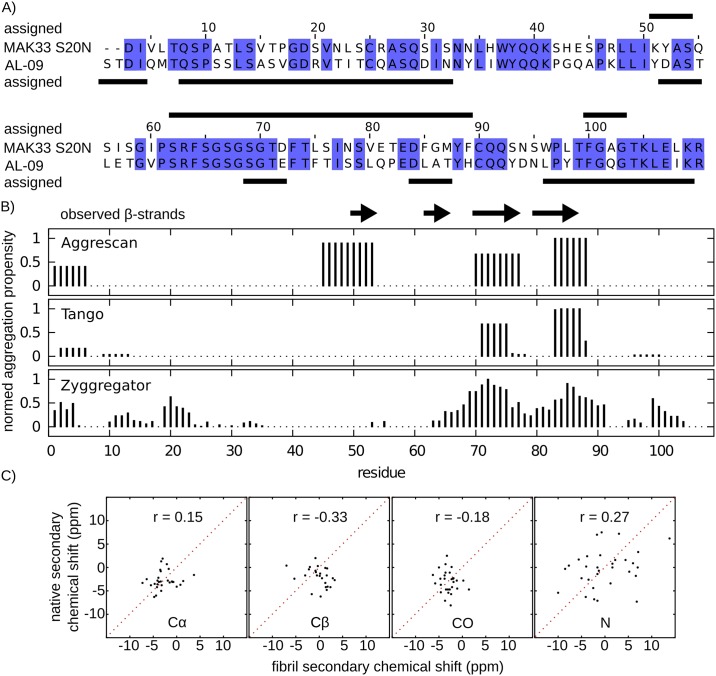
Comparison with AL-09, amyloid prediction algorithms and native state chemical shifts. A) Sequence alignment of MAK33 V_L_ S20N and AL-09 V_L_: Identical residues are marked in blue. Residues assigned in MAS ssNMR spectra are indicated by bars above and below the corresponding sequence. B) Predictions of MAK33 V_L_ S20N amyloid propensity and experimentally observed β-strands. C) Secondary chemical shift correlation of MAK33 V_L_ S20N in the solid-state (fibrils, pH 2) and solution-state (native, pH 6.5) for Cα, Cβ, CO and N chemical shifts. The cross-correlation coefficients r are indicated in each plot.

In the AL-09 fibril sample studied by Piehl et al. [18,19], the structured regions comprised mostly the first 30 N-terminal residues as well as residues 94–107 at the C-terminus. In between, only a few residues could be assigned. It is surprising that the assigned regions differ considerably compared to our sequence (Fig 4A). In both studies, several residues could not be assigned sequentially, so there might be more overlap in the hydrophobic core regions. We emphasise however, that intense resonances, which are easier to assign, correlate with rigid structure. In this regard, the differences with respect to assigned regions are meaningful and indicate distinct topologies for the fibril states of both V_L_ sequences. Recently, LC amyloid deposits with distinct morphologies were found in *ex vivo* tissue samples of one individual patient [42]. In this sense, the V_L_ fibril structure is polymorphic due to growth conditions and tissue specific factors, in addition to the structural diversity which is presumably induced by differences in the sequence.

Piehl et al. have previously pointed out that most of the non-conservative mutations in AL-09 are located outside of the assigned and thus structured regions [18]. Similarly, the amyloid-enhancing mutation S20N employed here for MAK33 is not part of the hydrophobic core identified in our study. These findings emphasise that point mutations, which are relevant for fibril formation, are not necessarily structured in the fibril state. On the other hand, the critical residue D70, which is part of a conserved salt bridge is located within the assigned regions [25]. Similarly, the highly amyloidogenic V_L_-λ6 subtype differs from less amyloidogenic variants by an insertion at positions 68 and 69 [43,44], which, according to our findings, is located in the fibril core. Understanding of amyloidogenicity of antibody LC sequences cannot be complete without considering the fibril structure and requires further ssNMR studies.

In this study, we investigated a murine VL sequence. To find out if the murine framework is comparable to human pathological sequences, we compared the MAK VL with human VL sequences. The sequence identity between MAK33 VL and other patient-derived or patient-related sequences ranges from 40% to 60%. E. g. the sequence identity is 57% with respect to Sma [45], 44% in comparison to 6aJL2 [46], and 42% for Wil [47]. While these values might seem low, they are actually in the same order as the identities between different pathological human sequences: The identity between Sma and 6aJL2 is 46%, 45% for Sma and Wil, and 89% for the quite similar λ6 sequences 6aJL2 and Wil. Hence, the differences between our murine sequence and pathological sequences are not larger than the differences between patient-derived AL sequences of different light chain subgroups.

We prepared our fibrils deliberately at pH 2, since acidic conditions are known to stabilise fibrils. pH 2 is necessary to form stable oligomers. Recently, ssNMR experiments have been performed to identify the structured core residues in the VL-κ protein AL-09 fibril. Also in this study, fibrils were prepared at pH 2 [18,19]. Piehl et al compared those *in vitro* fibrils formed at acidic conditions with *ex vivo* fibrils derived from human spleen tissue. It is discussed that NMR spectra from both samples are comparable. Hence, while acidic conditions are not physiological, they represent a necessary experimental requirement for the investigation of fibrillised VL protein by ssNMR.

The vast number of different V_L_ sequences that can form fibrils makes a structural characterisation of all antibody fibrils by ssNMR impractical. Therefore, predictions of amyloidogenic regions can be a precious tool to study a multitude of sequences. We compared our MAS ssNMR results with the predictions from three web tools (Fig 4B). In our benchmark, we included Aggrescan [48], Tango [49] and Zyggregator [50]. Of the four β-strands identified by MAS ssNMR, all algorithms identified the two longer C-terminal β-strands between residues 70 and 90. Our first N-terminal β-strand was only predicted by Aggrescan, the second one only with low propensity by Zyggregator. All programs predicted moderate aggregation propensities in the N-terminal region (residues 1–30), where we could not assign any resonances. Our assignments are incomplete and we cannot exclude the presence of further structured regions. In fact, a structured N-terminus of V_L_ fibrils has also been suggested for other V_L_ sequences [15,16]. To summarise, all three algorithms could correctly predict the two longer β-strands. We therefore believe that prediction of amyloid propensity will be a valuable tool to study VL sequences in the future.

In the past, domain swapping has been suggested as a mechanism to explain immunoglobulin light chain deposition [51,52]. In this context, it has been shown that the amyloid core of transthyretin consists of native-like β-sheets [53]. We analysed NMR secondary chemical shifts, which report on secondary structure elements. If the conformation of MAK33 V_L_ S20N in the fibril state resembled the conformation of this point mutant in the native structure, the secondary chemical shifts of the solid-state and solution-state preparations should be similar. Fig 4C shows correlation plots of the Cα, Cβ, CO and N secondary chemical shifts. We can not observe a correlation between native and fibril secondary structure for any of the nuclei assigned in both conformations. Hence, we conclude the fibrils studied here were not formed by domain swapping. Similar discrepancies between native and fibril states were reported recently for AL-09 V_L_ [19], supporting the hypothesis that AL fibrils do not form via a domain-swapping mechanism.

Our data alternatively indicate that the native MAK33 V_L_ at least partially unfolds and undergoes a transformation to a distinct oligomeric state. While the morphology according to TEM differs from mature fibrils, the local chemical environment is rather similar, as reflected by the NMR chemical shifts. Along the same lines, NMR spectra for oligomer and protofibril preparations have been reported for amyloid-β [54]. Our FTIR experiment with the oligomers displayed a characteristic peak at 1697 cm^-1^. While there are still disputes about determination of β-sheet arrangements with FTIR [35], according to theoretical derivations, this band might indicate anti-parallel β-sheets [55]. The fibrils, in contrast, do not produce this FTIR peak and thus likely contain the typical parallel β-sheets [56]. Different orientations of β-strands in oligomers and fibrils are in agreement with literature [35,57,58]. It will be interesting to see, whether these observations on MAK33 V_L_ oligomers and fibrils are also true for other light chain amyloid sequences.

With the recent advances in MAS ssNMR structure determinations of fibrils [59,60] and the now available assignments of V_L_ fibrils, a structure comes within reach. We expect this will be an important contribution to predict which V_L_ sequences are prone to fibril formation and how the oligomers and fibrils can be cleared.

## Material and methods

If not specified otherwise, all chemicals were purchased from Sigma Aldrich (Taufkirchen, Germany).

### Recombinant protein production

MAK33 V_L_ WT and S20N were purified as described previously [25]. Briefly, *E*.*coli* BL21 with a pET28 vector containing the MAK V_L_ gene were grown in M9_Kan_ minimal medium supplemented with 2 g/l ^13^C-glucose and 0.5 g/l ^15^NH_4_Cl. Expression was induced with 1 mM IPTG at OD 0.6–0.8. After over night expression at 37°C, cells were harvested and inclusion bodies were isolated. The inclusion bodies were dissolved in a buffer consisting of 50 mM Tris, 5 mM EDTA, 8 M urea and 1% β-mercaptoethanol (pH 8). The dissolved protein was subjected to anion exchange chromatography, using a 16 / 10 Q Sepharose High Performance column (GE Healthcare, Munich, Germany) using a buffer consisting of 25 mM Tris, 5 mM EDTA and 5 M urea (pH 8). The V_L_ protein eluted in the flowthrough. Refolding was achieved by over-night dialysis into a buffer containing 250 mM Tris, 5 mM EDTA, 100 mM L-arginine, 1mM oxidised glutathione und 0.5 mM reduced glutathione (pH 8, 4°C). Finally, the protein was purified using gel filtration chromatography with a HiLoad 16/600 Superdex 75 prep grade column (GE Healthcare, Munich, Germany) and a buffer consisting of 20 mM sodium phosphate and 50 mM NaCl (pH 6.5). Total yield was 15–20 mg protein per liter of culture.

### Oligomer and fibril preparation

The buffer was thoroughly exchanged to 25 mM acetic acid, 25 mM phosphoric acid and 50 mM NaCl (pH 2). Protein monomer concentration was set to 50 μM. 0.05% NaN_3_ was supplemented to protect against bacterial growth. In order to obtain non-fibrillar oligomers, the solution was incubated at room temperature under quiescent conditions. For fibril formation, the solution was incubated at 37°C at 350 rpm. Fibrils formed within one week. The outcome was confirmed by TEM. To enrich a single polymorph, fibrils were seeded for several generations. Approximately 2% of preformed fibrils were added to a new batch set up for fibril formation. Samples used for MAS ssNMR analysis were from 7^th^ or later generation of continuous seeding, with each seeding step incubating for one week.

### MAS solid-state NMR sample preparation

An OptimaL-100 XP ultracentrifuge (Beckman Coulter, Krefeld, Germany) equipped with an SW 32 Ti swinging bucket rotor and a rotor filling device (Giotto Biotech, Florence, Italy) were used to pack the protein aggregate into an MAS rotor. The rotation frequency of the centrifuge was set to 28,000 rpm. Samples were packed into 3.2 mm thin wall ZrO_2_ rotors with vespel caps (CortecNet, Voisins Le Bretonneux, France) employing house-made teflon spacers.

### MAS solid-state NMR experiments

All MAS ssNMR experiments were recorded using a 750 MHz Bruker Avance III spectrometer (Bruker BioSpin, Karlsruhe) equipped with a triple-resonance MAS probe for 3.2 mm rotors. The set temperature of the probe was adjusted to 273 K. The MAS frequency was set to 10 kHz (for PDSD, NCACX, NCOCX) or 17 kHz (NCOCA, CANCO, NCACB, NCOCAB, INEPT). Detailed settings for the experiments are given in the Supplementary Information (S1 Table). The building blocks for the experiments were PDSD, SPECIFIC-CP [61], BSH-CP [62], DREAM [63] and INEPT. The pulse sequences were implemented as described by Szeverenyi et al. [29] and Schütz et al. [31]. NCACX, NCOCX and NCACB were acquired with non-uniform sampling to increase sensitivity [64]. Sparsity was set to 50% with exponentially decreasing sampling density, matched to the transverse relaxation time according to a spin echo experiment [65]. Experiments were acquired using Topspin 3.2 (Bruker BioSpin, Karlsruhe, Germany). Uniformly sampled experiments were processed with Topspin 3.2, nonuniformly sampled spectra were processed either with the compressed sensing plugin of Topspin 3.2 or with hmsIST [66] in combination with NMRPipe [67]. All spectra were zero-filled to double the number of measured points and then the next power of two. Shifted squared cosine functions were employed for apodisation. Secondary chemical shifts were calculated as

$$\Delta \delta \;=\;\delta^{observed}–\;\delta^{random\;coil}$$

with random coil chemical shifts taken from the BMRB [68].

### Negative-stain transmission electron microscopy

Copper grids with 300 meshes coated with formvar/carbon film (Electron Microscopy Sciences, Hatfield, USA) were glow-discharged in argon atmosphere for 30 s at 3 mA. 5 μl of a 50 μM protein sample were incubated for 60 s on the grid. After removing the protein solution, the grid was washed with water. 5 μl uranyl acetate solution (2% w/v) were applied on the grid for staining and removed after 30 s. Photographs of the oligomers and fibrils converted from oligomers were measured on a Jeol JEM 1400 Plus transmission electron microscope (Jeol, Tokyo, Japan).

### Fourier-transform infrared spectroscopy

Oligomer and fibril samples, 50 μM in fibril buffer (25 mM phosphoric acid, 25 mM acetic acid, 50 mM NaCl, 0.05% NaN_3_, pH 2), were recorded on a JASCO FT/IR-4100 FT-IR spectrometer (JASCO, Gross-Umstadt, Germany) with attenuated total reflectance (ATR) attachment. The samples were measured with 128 scans at a resolution of 2 cm^-1^ at room temperature. Spectra were buffer subtracted and smoothed using a Savitzky-Golan algorithm.

## Supporting information

S1 Fig Sequential walks from residues G64 to G68. (PDF)

S2 Fig Secondary chemical shifts of MAK33 V_L_ S20N fibrils. (PDF)

S3 Fig ^1^H-^15^N-INEPT experiment of MAK33 V_L_ S20N fibrils. (PDF)

S4 Fig Electron microscopic analysis of MAK33 V_L_ S20N oligomers. (PDF)

S1 Table ssNMR parameters. (XLS)

## References

[1] Desport E, Bridoux F, Sirac C, Delbes S, Bender S, Fernandez B, et al AL Amyloidosis. Orphanet J Rare Dis. 2012;7(54). 10.1186/1750-1172-7-54 PMC3495844 22909024

[2] Ramirez-Alvarado M. Amyloid Formation in Light Chain Amyloidosis. Curr Top Med Chem. 2012;12(22):2523–33. 23339305 10.2174/1568026611212220007 PMC3606678

[3] Merlini G, Seldin DC, Gertz MA. Amyloidosis: Pathogenesis and New Therapeutic Options. J Clin Oncol. 2011;29(14):1924–33. doi: 10.1200/JCO.2010.32.2271 21483018 10.1200/JCO.2010.32.2271 PMC3138545

[4] Huang X, Wang Q, Jiang S, Chen W, Zeng C, Liu Z. The clinical features and outcomes of systemic AL amyloidosis: a cohort of 231 Chinese patients. Clin Kidney J. 2015 2;8(1):120–6. doi: 10.1093/ckj/sfu117 25713722 10.1093/ckj/sfu117 PMC4310422

[5] Kumar SK, Gertz MA, Lacy MQ, Dingli D, Hayman SR, Buadi FK, et al Recent Improvements in Survival in Primary Systemic Amyloidosis and the Importance of an Early Mortality Risk Score. Mayo Clin Proc. 2011;86(1):12–8. doi: 10.4065/mcp.2010.0480 21193650 10.4065/mcp.2010.0480 PMC3012628

[6] Breydo L, Uversky VN. Structural, morphological, and functional diversity of amyloid oligomers. FEBS Lett. 2015;589(19):2640–8. 26188543 10.1016/j.febslet.2015.07.013

[7] Tipping KW, Karamanos TK, Jakhria T, Iadanza MG, Goodchild SC, Tuma R, et al pH-induced molecular shedding drives the formation of amyloid fibril-derived oligomers. Proc Natl Acad Sci. 2015;112(18):5691–6. doi: 10.1073/pnas.1423174112 25902516 10.1073/pnas.1423174112 PMC4426459

[8] Haass C, Selkoe DJ. Soluble protein oligomers in neurodegeneration: lessons from the Alzheimer’s amyloid beta-peptide. Nat Rev Mol Cell Biol. 2007;8(2):101–12. doi: 10.1038/nrm2101 17245412 10.1038/nrm2101

[9] Campioni S, Mannini B, Zampagni M, Pensalfini A, Parrini C, Evangelisti E, et al A causative link between the structure of aberrant protein oligomers and their toxicity. Nat Chem Biol. 2010;6(2):140–7. doi: 10.1038/nchembio.283 20081829 10.1038/nchembio.283

[10] Lashuel HA, Lansbury PT. Are amyloid diseases caused by protein aggregates that mimic bacterial pore-forming toxins? Q Rev Biophys. 2006;39(2):167–201. doi: 10.1017/S0033583506004422 16978447 10.1017/S0033583506004422

[11] Fändrich M. Oligomeric intermediates in amyloid formation: structure determination and mechanisms of toxicity. J Mol Biol. 2012;421(4–5):427–40. doi: 10.1016/j.jmb.2012.01.006 22248587 10.1016/j.jmb.2012.01.006

[12] Xue W-F, Hellewell AL, Gosal WS, Homans SW, Hewitt EW, Radford SE. Fibril fragmentation enhances amyloid cytotoxicity. J Biol Chem. 2009 12 4;284(49):34272–82. doi: 10.1074/jbc.M109.049809 19808677 10.1074/jbc.M109.049809 PMC2797196

[13] McWilliams-Koeppen HP, Foster JS, Hackenbrack N, Ramirez-Alvarado M, Donohoe D, Williams A, et al Light Chain Amyloid Fibrils Cause Metabolic Dysfunction in Human Cardiomyocytes. PLoS One. 2015;10(9):e0137716 doi: 10.1371/journal.pone.0137716 26393799 10.1371/journal.pone.0137716 PMC4579077

[14] Marin-Argany M, Lin Y, Misra P, Williams A, Wall J, Howell K, et al Cell Damage in Light Chain Amyloidosis: Fibril Internalization, Toxicity and Cell-Mediated Seeding. J Biol Chem. 2016;291(38):19813–25. doi: 10.1074/jbc.M116.736736 27462073 10.1074/jbc.M116.736736 PMC5025671

[15] O’Nuallain B, Allen A, Kennel SJ, Weiss DT, Solomon A, Wall JS. Localization of a Conformational Epitope Common to Non-Native and Fibrillar Immunoglobulin Light Chains. Biochemistry. 2007;46(5):1240–7. doi: 10.1021/bi0616605 17260953 10.1021/bi0616605 PMC1832162

[16] Del Pozo-Yauner L, Wall JS, González Andrade M, Sánchez-López R, Rodríguez-Ambriz SL, Pérez Carreón JI, et al The N-terminal strand modulates immunoglobulin light chain fibrillogenesis. Biochem Biophys Res Commun. 2014;443(2):495–9. doi: 10.1016/j.bbrc.2013.11.123 24321098 10.1016/j.bbrc.2013.11.123

[17] Schmidt A, Annamalai K, Schmidt M, Grigorieff N, Fändrich M. Cryo-EM reveals the steric zipper structure of a light chain-derived amyloid fibril. Proc Natl Acad Sci. 2016;113(22):6200–5. doi: 10.1073/pnas.1522282113 27185936 10.1073/pnas.1522282113 PMC4896715

[18] Piehl DW, Blancas-Mejia LM, Ramirez-Alvarado M, Rienstra CM. Solid-state NMR chemical shift assignments for AL-09 VL immunoglobulin light chain fibrils. Biomol NMR Assign. 2016;11(1):45–50. doi: 10.1007/s12104-016-9718-3 27771830 10.1007/s12104-016-9718-3 PMC5344749

[19] Piehl DW, Blancas-Mejía LM, Wall JS, Kennel SJ, Ramirez-Alvarado M, Rienstra CM. Immunoglobulin Light Chains Form an Extensive and Highly Ordered Fibril Involving the N- and C-Terminus. ACS Omega. 2017;2(2):712–20. doi: 10.1021/acsomega.6b00494 28261692 10.1021/acsomega.6b00494 PMC5331457

[20] Augustine JG, de La Calle A, Knarr G, Buchner J, Frederick CA. The crystal structure of the Fab fragment of the monoclonal antibody MAK33. J Biol Chem. 2001;276(5):3287–94. doi: 10.1074/jbc.M005221200 11036070 10.1074/jbc.M005221200

[21] Simpson ER, Herold EM, Buchner J. The folding pathway of the antibody V(L) domain. J Mol Biol. 2009 10;392(5):1326–38. doi: 10.1016/j.jmb.2009.07.075 19647749 10.1016/j.jmb.2009.07.075

[22] Feige MJ, Simpson ER, Herold EM, Bepperling A, Heger K, Buchner J. Dissecting the alternatively folded state of the antibody Fab fragment. J Mol Biol. 2010;399(5):719–30. doi: 10.1016/j.jmb.2010.04.032 20434459 10.1016/j.jmb.2010.04.032

[23] Nokwe CN, Zacharias M, Yagi H, Hora M, Reif B, Goto Y, et al A Residue-specific Shift in Stability and Amyloidogenicity of Antibody Variable Domains. J Biol Chem. 2014;289(39):26829–46. doi: 10.1074/jbc.M114.582247 25096580 10.1074/jbc.M114.582247 PMC4175325

[24] Nokwe CN, Hora M, Zacharias M, Yagi H, John C, Reif B, et al The Antibody Light-Chain Linker Is Important for Domain Stability and Amyloid Formation. J Mol Biol. 2015;427(22):3572–86. doi: 10.1016/j.jmb.2015.09.012 26408269 10.1016/j.jmb.2015.09.012

[25] Nokwe CN, Hora M, Zacharias M, Yagi H, Peschek J, Reif B, et al A Stable Mutant Predisposes Antibody Domains to Amyloid Formation through Specific Non-Native Interactions. J Mol Biol. 2016;428(6):1315–32. doi: 10.1016/j.jmb.2016.01.015 26827727 10.1016/j.jmb.2016.01.015

[26] Hora M, Carballo-Pacheco M, Weber B, Morris VK, Wittkopf A, Buchner J, et al Epigallocatechin-3-gallate preferentially induces aggregation of amyloidogenic immunoglobulin light chains. Sci Rep. 2017;7(41515). 10.1038/srep41515 PMC5269747 28128355

[27] Higman VA, Flinders J, Hiller M, Jehle S, Markovic S, Fiedler S, et al Assigning large proteins in the solid state: a MAS NMR resonance assignment strategy using selectively and extensively 13C-labelled proteins. J Biomol NMR. 2009;44(4):245–60. doi: 10.1007/s10858-009-9338-7 19609683 10.1007/s10858-009-9338-7

[28] Toyama BH, Weissman JS. Amyloid structure: conformational diversity and consequences. Annu Rev Biochem. 2011;80:557–85. doi: 10.1146/annurev-biochem-090908-120656 21456964 10.1146/annurev-biochem-090908-120656 PMC3817101

[29] Szeverenyi NM, Sullivan MJ, Maciel GE. Observation of spin exchange by two-dimensional fourier transform 13C cross polarization-magic-angle spinning. J Magn Reson. 1982 5;47(3):462–75.

[30] Pauli J, Baldus M, van Rossum B, de Groot H, Oschkinat H. Backbone and Side-Chain 13C and 15N Signal Assignments of the α-Spectrin SH3 Domain by Magic Angle Spinning Solid-State NMR at 17.6 Tesla. ChemBioChem. 2001;2(4):272–81. 11828455 10.1002/1439-7633(20010401)2:4<272::AID-CBIC272>3.0.CO;2-2

[31] Schuetz A, Wasmer C, Habenstein B, Verel R, Greenwald J, Riek R, et al Protocols for the sequential solid-state NMR spectroscopic assignment of a uniformly labeled 25 kDa protein: HET-s(1–227). Chembiochem. 2010;11(11):1543–51. doi: 10.1002/cbic.201000124 20572250 10.1002/cbic.201000124

[32] Shen Y, Delaglio F, Cornilescu G, Bax A. TALOS+: A hybrid method for predicting protein backbone torsion angles from NMR chemical shifts. J Biomol NMR. 2009;44(4):213–23. doi: 10.1007/s10858-009-9333-z 19548092 10.1007/s10858-009-9333-z PMC2726990

[33] Zandomeneghi G, Krebs MRH, Mccammon MG, Fändrich M. FTIR reveals structural differences between native beta-sheet proteins and amyloid fibrils. Protein Sci. 2004;13(12):3314–21. doi: 10.1110/ps.041024904 15537750 10.1110/ps.041024904 PMC2287307

[34] Ami D, Lavatelli F, Rognoni P, Palladini G, Raimondi S, Giorgetti S, et al In situ characterization of protein aggregates in human tissues affected by light chain amyloidosis: a FTIR microspectroscopy study. Sci Rep. 2016;6(29096). 10.1038/srep29096 PMC4931462 27373200

[35] Sarroukh R, Goormaghtigh E, Ruysschaert J-M, Raussens V. ATR-FTIR: A “rejuvenated” tool to investigate amyloid proteins. Biochim Biophys Acta—Biomembr. 2013;1828(10):2328–38. 10.1016/j.bbamem.2013.04.012 23746423

[36] Mainz A, Jehle S, van Rossum BJ, Oschkinat H, Reif B. Large protein complexes with extreme rotational correlation times investigated in solution by magic-angle-spinning NMR spectroscopy. J Am Chem Soc. 2009 11 11;131(44):15968–15969. doi: 10.1021/ja904733v 19839609 10.1021/ja904733v

[37] Schubert M, Labudde D, Oschkinat H, Schmieder P. A software tool for the prediction of Xaa-Pro peptide bond conformations in proteins based on 13C chemical shift statistics. J Biomol NMR. 2002 10;24(2):149–54. 12495031 10.1023/a:1020997118364

[38] Lang K, Schmid FX, Fischer G. Catalysis of protein folding by prolyl isomerase. Nature. 1987;329:268–70. doi: 10.1038/329268a0 3306408 10.1038/329268a0

[39] Lilie H, Lang K, Rudolph R, Buchner J. Prolyl isomerases catalyze antibody folding in vitro. Protein Sci. 1993 9;2(9):1490–6. doi: 10.1002/pro.5560020913 8104614 10.1002/pro.5560020913 PMC2142458

[40] Jahn TR, Parker MJ, Homans SW, Radford SE. Amyloid formation under physiological conditions proceeds via a native-like folding intermediate. Nat Struct Mol Biol. 2006 3;13(3):195–201. doi: 10.1038/nsmb1058 16491092 10.1038/nsmb1058 PMC7617781

[41] Blancas-Mejía LM, Tischer A, Thompson JR, Tai J, Wang L, Auton M, et al Kinetic Control in Protein Folding for Light Chain Amyloidosis and the Differential Effects of Somatic Mutations. J Mol Biol. 2014 1 23;426(2):347–61. doi: 10.1016/j.jmb.2013.10.016 24157440 10.1016/j.jmb.2013.10.016 PMC3892967

[42] Annamalai K, Gührs K-H, Koehler R, Schmidt M, Michel H, Loos C, et al Polymorphism of Amyloid Fibrils In Vivo. Angew Chemie Int Ed. 2016;55(15):4822–5. 10.1002/anie.201511524 PMC4864496 26954430

[43] Dwulet FE, Strako K, Benson MD. Amino Acid Sequence of a lambda VI Primary (AL) Amyloid Protein (WLT). Scand J Immunol. 1985 12;22(6):653–60. 4089539 10.1111/j.1365-3083.1985.tb01927.x

[44] Bellotti V, Mangione P, Merlini G. Review: Immunoglobulin Light Chain Amyloidosis—The Archetype of Structural and Pathogenic Variability. J Struct Biol. 2000 6;130(2):280–9. 10940232 10.1006/jsbi.2000.4248

[45] Stevens PW, Raffen R, Hanson DK, Deng YL, Berrios-Hammond M, Westholm FA, et al Recombinant immunoglobulin variable domains generated from synthetic genes provide a system for in vitro characterization of light-chain amyloid proteins. Protein Sci. 1995 3;4(3):421–32. doi: 10.1002/pro.5560040309 7795526 10.1002/pro.5560040309 PMC2143084

[46] del P Yauner L, Ortiz E, Sanchez R, Sanchez-Lopez R, Güereca L, Murphy CL, et al Influence of the germline sequence on the thermodynamic stability and fibrillogenicity of human lambda 6 light chains. Proteins. 2008;72(2):684–92. doi: 10.1002/prot.21934 18260098 10.1002/prot.21934

[47] Pokkuluri PR, Solomon A, Weiss DT, Stevens FJ, Schiffer M. Tertiary structure of human lambda 6 light chains. Amyloid. 1999;6(3):165–71. 10524280 10.3109/13506129909007322

[48] Conchillo-Solé O, de Groot NS, Avilés FX, Vendrell J, Daura X, Ventura S. AGGRESCAN: a server for the prediction and evaluation of “hot spots” of aggregation in polypeptides. BMC Bioinformatics. 2007;8(65). 10.1186/1471-2105-8-65 PMC1828741 17324296

[49] Fernandez-Escamilla A-M, Rousseau F, Schymkowitz J, Serrano L. Prediction of sequence-dependent and mutational effects on the aggregation of peptides and proteins. Nat Biotechnol. 2004 10;22(10):1302–6. doi: 10.1038/nbt1012 15361882 10.1038/nbt1012

[50] Tartaglia GG, Pawar AP, Campioni S, Dobson CM, Chiti F, Vendruscolo M. Prediction of aggregation-prone regions in structured proteins. J Mol Biol. 2008 7 4;380(2):425–36. doi: 10.1016/j.jmb.2008.05.013 18514226 10.1016/j.jmb.2008.05.013

[51] Bennett MJ, Sawaya MR, Eisenberg D. Deposition Diseases and 3D Domain Swapping. Structure. 2006;14(5):811–24. doi: 10.1016/j.str.2006.03.011 16698543 10.1016/j.str.2006.03.011

[52] Sonnen AFP, Yu C, Evans EJ, Stuart DI, Davis SJ, Gilbert RJC. Domain metastability: A molecular basis for immunoglobulin deposition? J Mol Biol. 2010;399(2):207–13. doi: 10.1016/j.jmb.2010.04.011 20394753 10.1016/j.jmb.2010.04.011 PMC2954335

[53] Lim KH, Dasari AKR, Hung I, Gan Z, Kelly JW, Wright PE, et al Solid-State NMR Studies Reveal Native-like β-sheet Structures in Transthyretin amyloid. Biochemistry. 2016;55(37):5272–8. doi: 10.1021/acs.biochem.6b00649 27589034 10.1021/acs.biochem.6b00649 PMC5035109

[54] Scheidt HA, Morgado I, Huster D. Solid-state NMR reveals a close structural relationship between amyloid-beta protofibrils and oligomers. J Biol Chem. 2012;287(27):22822–6. doi: 10.1074/jbc.M112.367474 22589542 10.1074/jbc.M112.367474 PMC3391088

[55] Chirgadze YN, Nevskaya NA. Infrared Spectra and Resonance Interaction of Amide-I Vibration of the Antiparallel-Chain Pleated Sheet. Biopolymers. 1976;15:607–25. doi: 10.1002/bip.1976.360150402 1252597 10.1002/bip.1976.360150402

[56] Chirgadze YN, Nevskaya NA. Infrared Spectra and Resonance Interaction of Amide-I Vibration of the Parallel-Chain Pleated Sheet. Biopolymers. 1976;15:627–36. doi: 10.1002/bip.1976.360150403 1252598 10.1002/bip.1976.360150403

[57] Cordeiro Y, Kraineva J, Suarez MC, Tempesta AG, Kelly JW, Silva JL, et al Fourier transform infrared spectroscopy provides a fingerprint for the tetramer and for the aggregates of transthyretin. Biophys J. 2006;91(3):957–67. doi: 10.1529/biophysj.106.085928 16698785 10.1529/biophysj.106.085928 PMC1563765

[58] Frare E, Mossuto MF, de Laureto PP, Tolin S, Menzer L, Dumoulin M, et al Characterization of Oligomeric Species on the Aggregation Pathway of Human Lysozyme. J Mol Biol. 2009;387(1):17–27. doi: 10.1016/j.jmb.2009.01.049 19361437 10.1016/j.jmb.2009.01.049

[59] Tuttle MD, Comellas G, Nieuwkoop AJ, Covell DJ, Berthold DA, Kloepper KD, et al Solid-state NMR structure of a pathogenic fibril of full-length human α-synuclein. Nat Struct Mol Biol. 2016;23(5):409–15. doi: 10.1038/nsmb.3194 27018801 10.1038/nsmb.3194 PMC5034296

[60] Aulikki Wälti M, Ravotti F, Arai H, Glabe CG, Wall JS, Böckmann A, et al Atomic-resolution structure of a disease-relevant Aβ(1–42) amyloid fibril. Proc Natl Acad Sci. 2016;113(34):4976–84. 27469165 10.1073/pnas.1600749113 PMC5003276

[61] Baldus M, Petkova AT, Herzfeld J, Griffin RG. Cross polarization in the tilted frame: assignment and spectral simplification in heteronuclear spin systems. Mol Phys. 1998;95(6):1197–207.

[62] Chevelkov V, Shi C, Fasshuber HK, Becker S, Lange A. Efficient band-selective homonuclear CO-CA cross-polarization in protonated proteins. J Biomol NMR. 2013 8;56(4):303–11. doi: 10.1007/s10858-013-9767-1 23925478 10.1007/s10858-013-9767-1

[63] Westfeld T, Verel R, Ernst M, Böckmann A, Meier BH. Properties of the DREAM scheme and its optimization for application to proteins. J Biomol NMR. 2012;53(2):103–12. doi: 10.1007/s10858-012-9627-4 22562365 10.1007/s10858-012-9627-4

[64] Palmer MR, Suiter CL, Henry GE, Rovnyak J, Hoch JC, Polenova T, et al Sensitivity of Nonuniform Sampling NMR. J Phys Chem B. 2015;119(22):6505–15. 10.1021/jp5126415 PMC4857715 25901905

[65] Hahn EL. Spin Echoes. Phys Rev. 1950;80(4):580–94.

[66] Hyberts SG, Milbradt AG, Wagner AB, Arthanari H, Wagner G. Application of Iterative Soft Thresholding for Fast Reconstruction of NMR Data Non-uniformly Sampled with Multidimensional Poisson Gap Scheduling. J Biomol NMR. 2012;52(4):315–27. doi: 10.1007/s10858-012-9611-z 22331404 10.1007/s10858-012-9611-z PMC3321367

[67] Delaglio F, Grzesiek S, Vuister GW, Zhu G, Pfeifer J, Bax A. NMRPipe: A multidimensional spectral processing system based on UNIX pipes. J Biomol NMR. 1995;6(3):277–93. 8520220 10.1007/BF00197809

[68] Ulrich EL, Akutsu H, Doreleijers JF, Harano Y, Ioannidis YE, Lin J, et al BioMagResBank. Nucleic Acids Res. 2008;36:402–8. 10.1093/nar/gkm957 PMC2238925 17984079

