# Supplementary material for: MAK33 antibody light chain amyloid fibrils are similar to oligomeric precursors
Source: PLoS One. 2017 Jul 26;12(7):e0181799. doi: 10.1371/journal.pone.0181799 (PMC5528828; doi:10.1371/journal.pone.0181799)
Supplement: S1 Fig — (PDF) [file pone.0181799.s001.pdf]

S1 Fig. Sequential walks from residues G64 to G68.

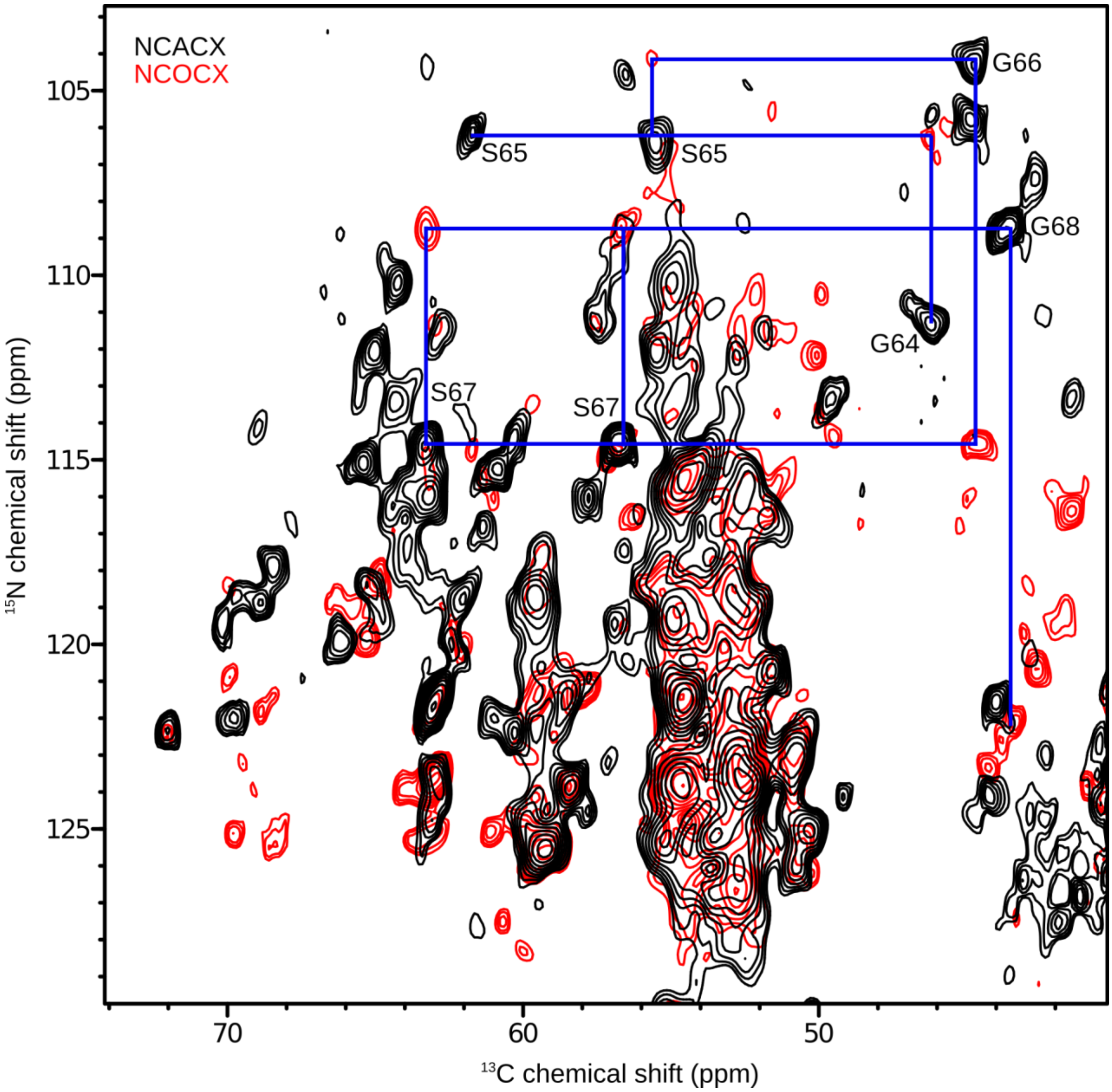

Sequential walk with 2D NCACX and NCOCX.

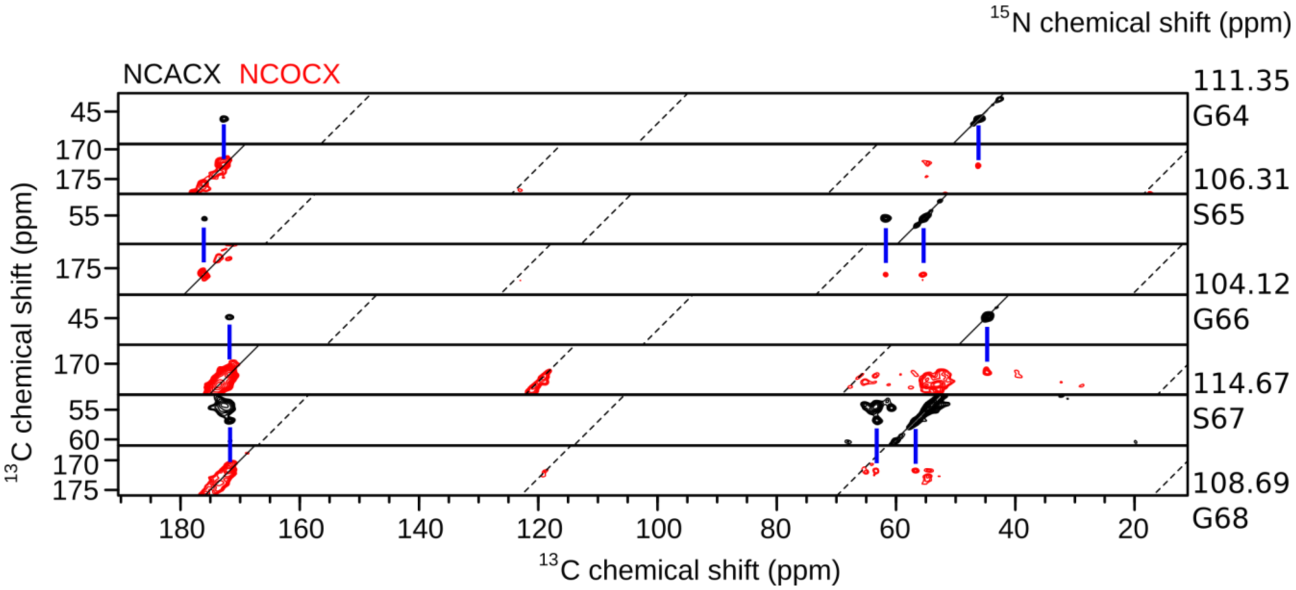

Sequential walk with 3D NCACX and NCOCX.

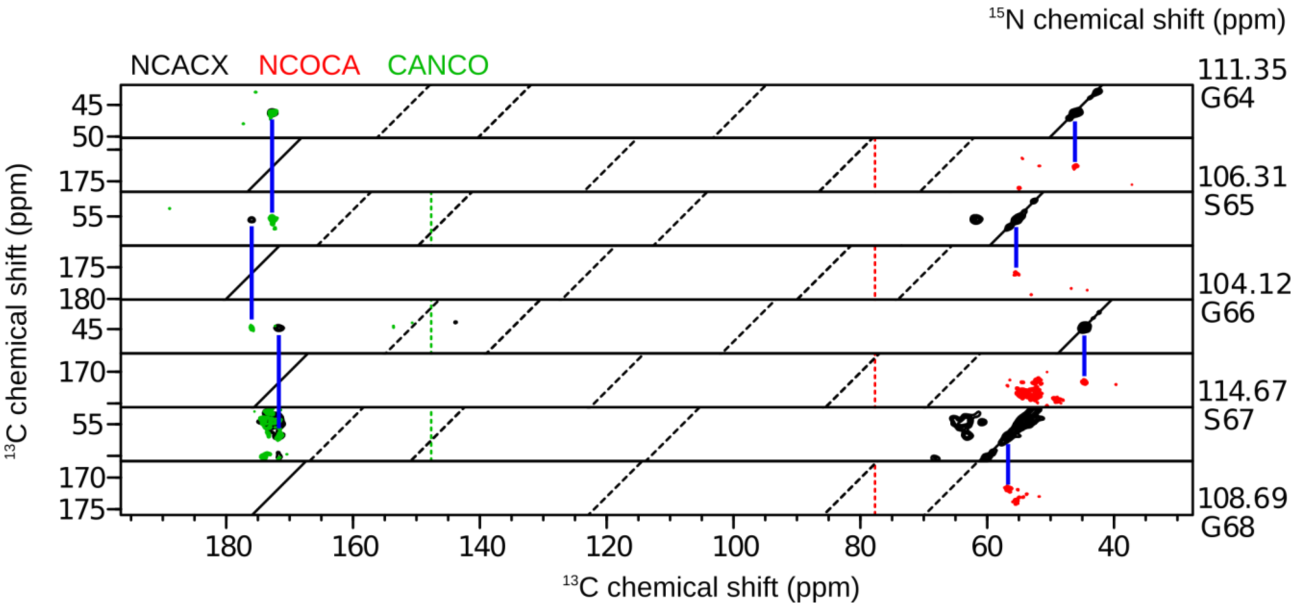

Sequential walk with 3D NCACX, NCOCA and CANCO.
