# Supplementary material for: MAK33 antibody light chain amyloid fibrils are similar to oligomeric precursors
Source: PLoS One. 2017 Jul 26;12(7):e0181799. doi: 10.1371/journal.pone.0181799 (PMC5528828; doi:10.1371/journal.pone.0181799)
Supplement: S2 Fig — (PDF) [file pone.0181799.s002.pdf]

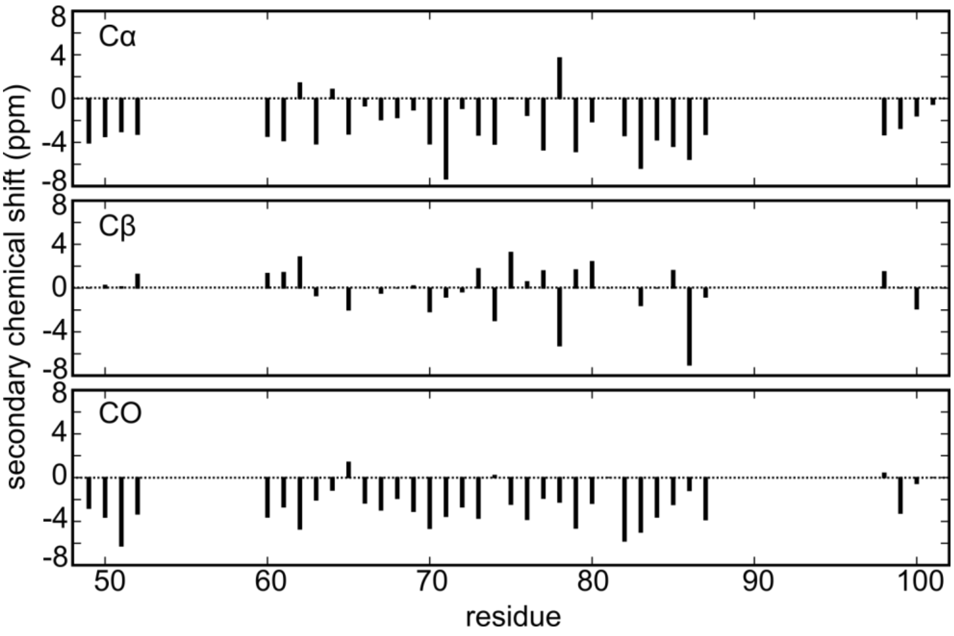

S2 Fig. Secondary chemical shifts of MAK33 V_L S20N fibrils.

The panels display the differences between observed and random coil chemical shifts for Cα, Cβ and CO. Negative Cα and CO secondary chemical shifts as well as positive Cβ secondary chemical shifts are characteristic for β-strands [1,2].

References:

1. Wishart DS, Sykes BD, Richards FM. Relationship between nuclear magnetic resonance chemical shift and protein secondary structure. J Mol Biol. 1991;222(2):311–33.

2. Spera S, Bax A. Empirical Correlation between Protein Backbone Conformation and Cα and Cβ 13C Nuclear Magnetic Resonance Chemical Shifts. J Am Chem Soc. 1991;113(14):5490–2.
