# Supplementary material for: MAK33 antibody light chain amyloid fibrils are similar to oligomeric precursors
Source: PLoS One. 2017 Jul 26;12(7):e0181799. doi: 10.1371/journal.pone.0181799 (PMC5528828; doi:10.1371/journal.pone.0181799)
Supplement: S3 Fig — (PDF) [file pone.0181799.s003.pdf]

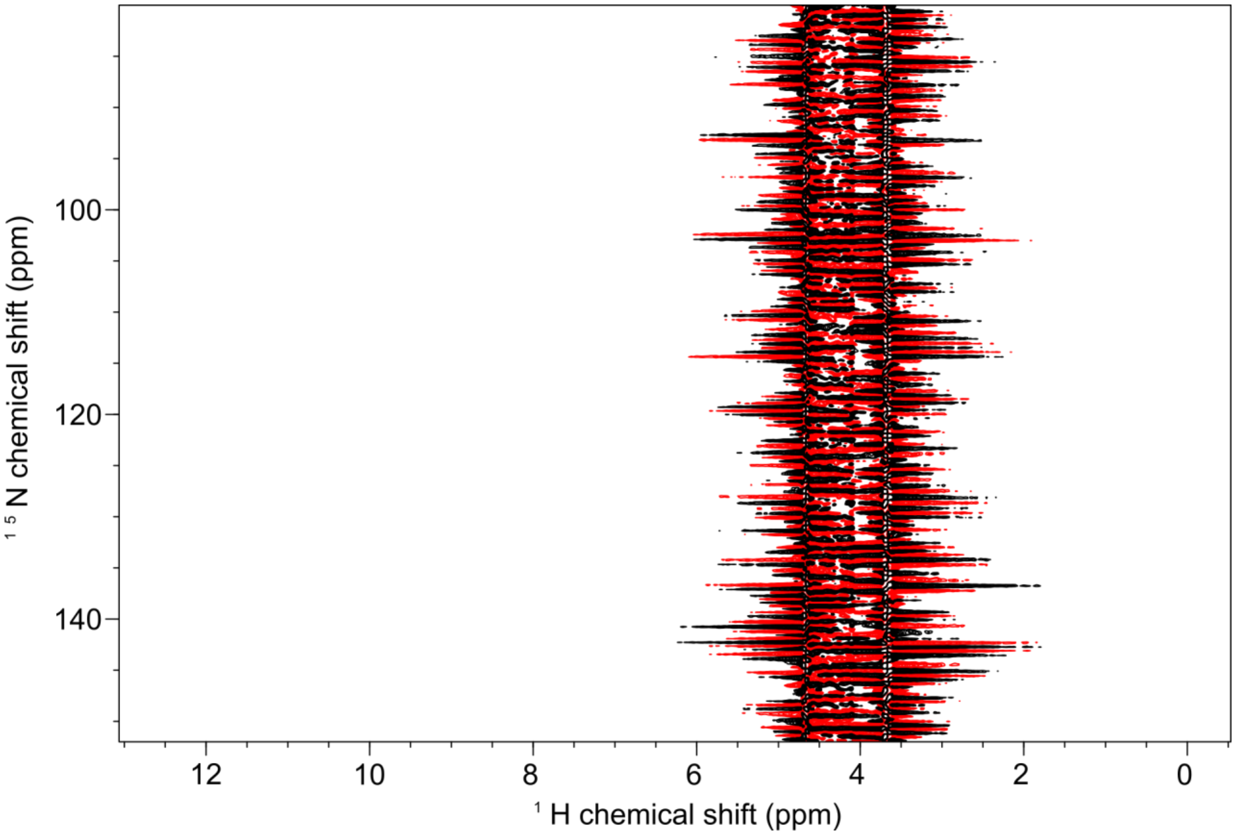

S3 Fig. ^{1}H-^{15}N-INEPT experiment of MAK33 V_L S20N fibrils.

No crosspeaks could be observed. Hence, those residues neither observed with PDSD nor with INEPT probably undergo intermediate dynamics, which are too fast for dipolar-coupling based experiments and too slow for INEPT. Alternatively, these residues might be structurally heterogeneous.
