# Supplementary material for: MAK33 antibody light chain amyloid fibrils are similar to oligomeric precursors
Source: PLoS One. 2017 Jul 26;12(7):e0181799. doi: 10.1371/journal.pone.0181799 (PMC5528828; doi:10.1371/journal.pone.0181799)
Supplement: S4 Fig — (PDF) [file pone.0181799.s004.pdf]

S4 Fig. Electron microscopic analysis of MAK33 V_L S20N oligomers.

particle #	Feret’s diameter (nm)
1	8.4
2	12.0
3	8.9
4	8.1
5	8.4
6	10.3
7	12.2
8	9.2
9	10.3
10	11.3
average	9.9
standard deviation	1.5
radius	5.0
volume	509.3 nm^3

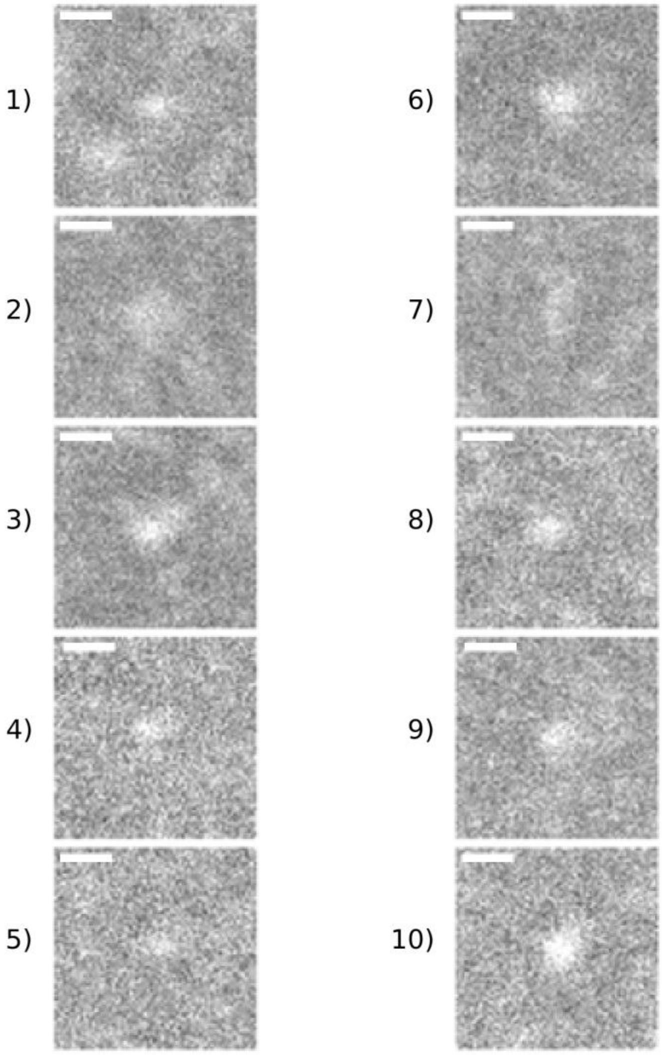

Particle diameters of the oligomers were measured as Feret’s statistical diameter [1], using the software ImageJ 1.50d (NIH, USA, https://imagej.nih.gov/ij/). The average diameter of the oligomers is 9.9 ± 1.5 nm, based on analysis of the 10 particles shown above. The scale bars in the images denote 10 nm.

Considering a mean diameter of 3.2 nm for MAK33 V_L in the native state (based on PDB ID 1FH5), the volume of the native state monomer is ca. 17.1 nm^3. Assuming that oligomer and monomer packing density are comparable, the volumes differ by a factor of 29.7, i.e. an oligomer consists of roughly 30 monomers.

According to previous analysis of the oligomers by analytical ultracentrifugation [2], most of the oligomers have a MW in the range of 200 to 500 kDa. Considering the monomeric MW of 12 kDa, this corresponds to 17 to 42 monomers per oligomer. This is in excellent agreement with our TEM result of ca. 30 monomers per oligomer.

References

1. Walton WH. Feret's statistical diameter as a measure of particle size. Nature. 1948;162:329–30.
2. Feige MJ, Simpson ER, Herold EM, Bepperling A, Heger K, Buchner J. Dissecting the alternatively folded state of the antibody Fab fragment. J Mol Biol. 2010;399(5):719–30.
